# Crystal structure of bis­(2-methyl-1*H*-imidazol-3-ium) tetra­chlorido­cobaltate(II)

**DOI:** 10.1107/S2056989015014127

**Published:** 2015-08-22

**Authors:** Mouhamadou Birame Diop, Libasse Diop, Thierry Maris

**Affiliations:** a Laboratoire de Chimie Minérale et Analytique, Département de Chimie, Faculté des Sciences et Techniques, Université Cheikh Anta Diop, Dakar, Sénégal; b Département de Chimie, Université de Montréal, 2900 Boulevard Édouard-Montpetit, Montréal, Québec, H3C 3J7, Canada

**Keywords:** crystal structure, cobalt complex, hydrogen bonds, organic–inorganic hybrid compound

## Abstract

The asymmetric unit of the title compound, (C_4_H_7_N_2_)_2_[CoCl_4_], consists of two 2-methyl­imidazolium cations and one tetra­hedral [CoCl_4_]^2−^ anion. The anions and cations inter­act through N—H⋯Cl hydrogen bonds to define layers with a stacking direction along [100]. Besides van der Waals forces, weak C—H⋯Cl inter­actions between these layers stabilize the crystal packing.

## Abstract 2

The tetra­hedral tetra­chlorido­cobaltate(II) anion is linked to bis­(2-methyl-1*H*-imidazol-3-ium) cations through N—H⋯Cl hydrogen bonds, resulting in a layered arrangement parallel to (100).

## Chemical context   

**Figure 1:**
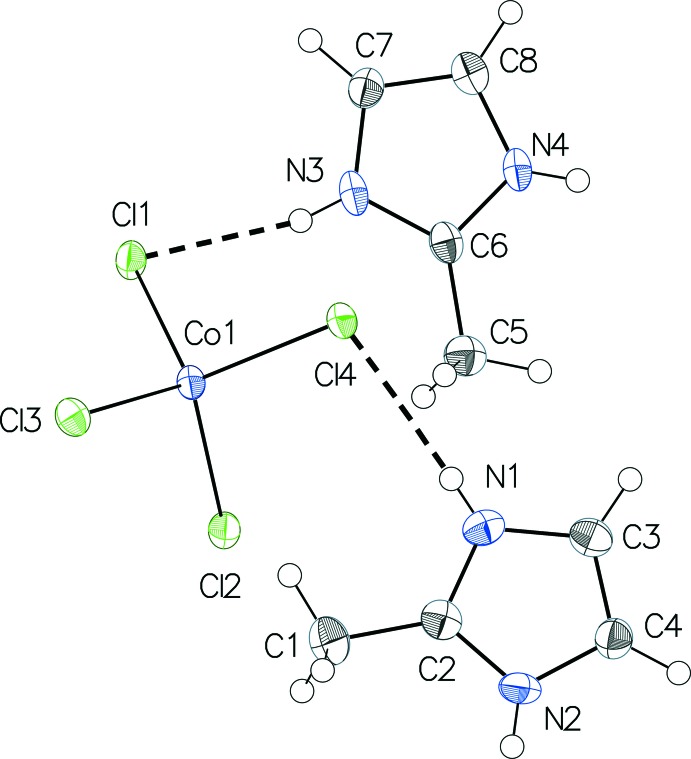
The mol­ecular components in the structure of the title compound, with displacement ellipsoids drawn at the 50% probability level. Hydrogen bonds of the N—H⋯Cl type are drawn as black dotted lines.

Studies of the behaviour of 2-methyl­imidazole as a ligand resulted in the title compound, (C_4_H_7_N_2_)_2_[CoCl_4_] (Fig. 1 ▸), which belongs to salts based on anionic metal halides. This family of organic–inorganic hybrid compounds has been studied intensively for its structural, thermal, spectroscopic and magnetic properties (Issaoui *et al.*, 2015 ▸). The structure of the related bis­(imidazolium) tetra­chlorido­cobaltate(II) salt has been reported by Zhang *et al.* (2005 ▸) (100 K data) and Adams *et al.* (2008 ▸) (298 K data).
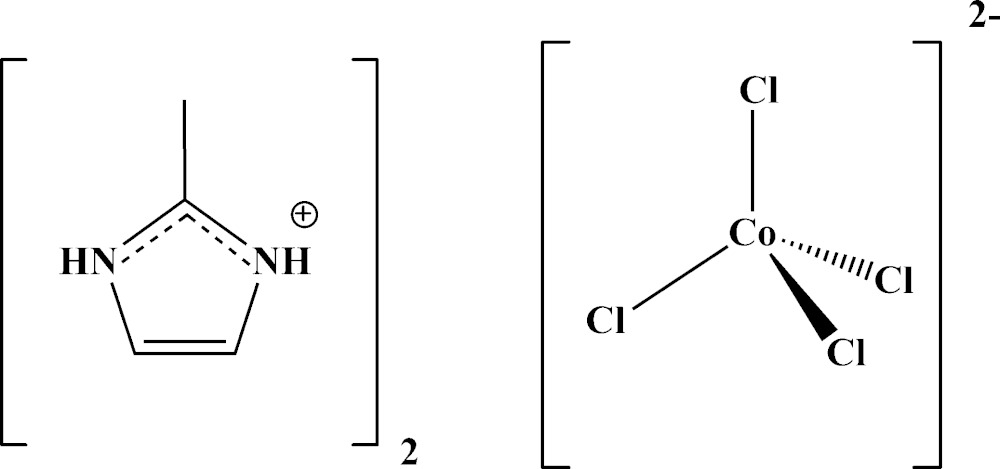


## Structural commentary   

The Co—Cl distances [2.2506 (8)–2.2907 (8) Å] are characteristic, and the mean distance (2.275 Å) is in very good agreement with the average Co—Cl bond length of 2.275 Å calculated on basis of 337 isolated [CoCl_4_]^2−^ anions from a set of 314 structures retrieved after a search in the Cambridge Structural Database (CSD, Version 5.36 with three updates; Groom & Allen, 2014 ▸). The longest Co—Cl distance in the title structure is observed for atom Cl4 which is an acceptor atom of two hydrogen bonds (Mghandef & Boughzala, 2015 ▸). The range for the Cl—Co—Cl angles [106.55 (3)–111.89 (3)°] indicates a slight distortion from the ideal tetra­hedral geometry. The imidazolium rings of the cations are planar with a maximum deviation of ±0.007 (2) Å and also are almost parallel to each other, with a dihedral angle between them of 0.9 (2)°. For the cations, the N—C distances involving the C atoms that carry the methyl groups (C2—N1/C2—N2 and C6—N3/C6—N4, respectively) are virtually the same (Table 1 ▸). A search in the CSD for 2-methyl­imidazolium cations returned 66 entries from 53 different structures. In 74% of them, these two distances differ by no more than 0.01 Å.

## Supra­molecular features   

**Figure 2:**
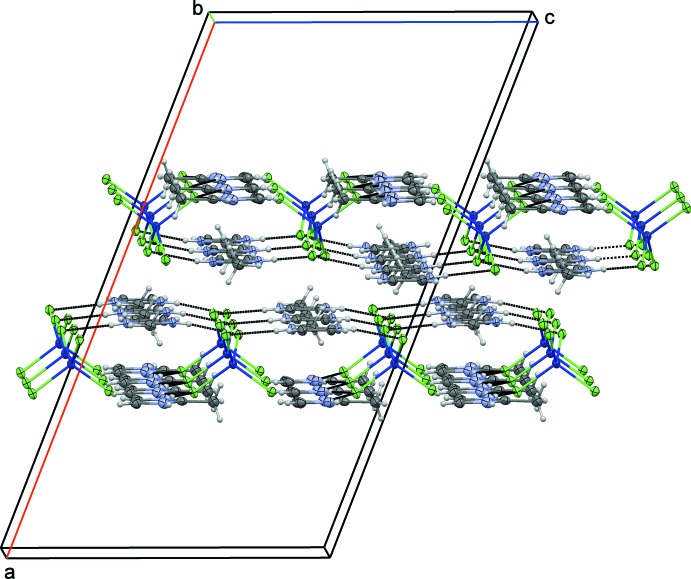
Partial packing diagram of the title structure viewed approximately along [010], showing two layers. Hydrogen bonds of the type N—H⋯Cl are drawn as black dotted lines.

The [CoCl_4_]^2−^ anion is linked *via* N—H⋯Cl hydrogen bonds to four cations and each cation is linked to two anions (Table 2 ▸). These inter­actions define layers parallel to (100) with alternating [CoCl_4_]^2−^ anions and cations (Fig. 2 ▸). Within these layers, the 2-methyl­imidazolium cations are involved in π–π stacking inter­actions with a centroid-to-centroid distance of 3.615 (2) Å and a distance between the mean planes of these rings of 3.340 (3) Å. Besides van der Waals forces, weak C—H⋯Cl inter­actions within and between the layers consolidate the crystal packing. The stacking direction of the layers is along [100].

## Synthesis and crystallization   

All starting materials were used as obtained without further purification. Methyl-2-imidazole and methyl­ammonium chloride were mixed in water with CoCl_2_·6H_2_O in an 1:2:1 ratio. Blue crystals suitable for single-crystal X-ray diffraction studies were obtained after slow solvent evaporation at room temperature.

## Refinement   

Crystal data, data collection and structure refinement details are summarized in Table 3 ▸. All H atoms were located from difference Fourier maps and were fully refined, except those that are part of the methyl group of the 2-methyl­imidazolium cations which were placed at calculated positions [C—H = 0.98 Å and *U*_iso_(H) = 1.5*U*_eq_(C)].

## Supplementary Material

Crystal structure: contains datablock(s) global, I. DOI: 10.1107/S2056989015014127/wm5189sup1.cif

Structure factors: contains datablock(s) I. DOI: 10.1107/S2056989015014127/wm5189Isup2.hkl

CCDC reference: 1415257

Additional supporting information:  crystallographic information; 3D view; checkCIF report

## Figures and Tables

**Table 1:** Selected bond lengths ()

N3C6	1.336(4)	Co1Cl1	2.2799(9)
N4C6	1.336(4)	Co1Cl2	2.2803(9)
N1C2	1.332(5)	Co1Cl3	2.2506(8)
N2C2	1.330(5)	Co1Cl4	2.2907(8)

**Table 2:** Hydrogen-bond geometry (, )

*D*H*A*	*D*H	H*A*	*D* *A*	*D*H*A*
N1H1Cl4	0.76(5)	2.46(5)	3.220(3)	177(5)
N2H2Cl4^i^	0.77(5)	2.54(5)	3.282(3)	163(4)
N3H3Cl1	0.79(4)	2.39(4)	3.166(3)	165(4)
N4H4Cl2^ii^	0.78(4)	2.42(5)	3.198(3)	175(4)
C4H4*A*Cl3^iii^	0.94(4)	2.73(4)	3.428(4)	132(3)
C3H3*A*Cl3^ii^	0.93(5)	2.70(5)	3.535(4)	151(4)
C8H8Cl1^iv^	0.96(5)	2.65(5)	3.575(4)	160(3)
C7H7Cl2^v^	0.94(4)	2.69(4)	3.617(4)	168(3)

Symmetry codes: (i) 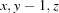; (ii) 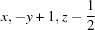; (iii) 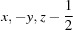; (iv) 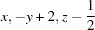; (v) 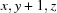.

**Table 3:** Experimental details

Crystal data	
Chemical formula	(C_4_H_7_N_2_)_2_[CoCl_4_]
*M*_r_	366.96
Crystal system, space group	Monoclinic, *C*2/*c*
Temperature (K)	100
*a*, *b*, *c* ()	26.847(3), 7.9029(8), 15.0938(14)
()	111.184(6)
*V* (^3^)	2986.0(5)
*Z*	8
Radiation type	Ga *K*, = 1.34139
(mm^1^)	10.45
Crystal size (mm)	0.23 0.12 0.06

Data collection	
Diffractometer	Bruker Venture Metaljet
Absorption correction	Multi-scan (*SADABS*; Krause *et al.*, 2015 ▸)
*T*_min_, *T*_max_	0.392, 0.752
No. of measured, independent and observed [*I* > 2(*I*)] reflections	27212, 3435, 3037
*R*_int_	0.063
(sin /)_max_ (^1^)	0.652

Refinement	
*R*[*F* ^2^ > 2(*F* ^2^)], *wR*(*F* ^2^), *S*	0.042, 0.102, 1.10
No. of reflections	3435
No. of parameters	188
H-atom treatment	H atoms treated by a mixture of independent and constrained refinement
_max_, _min_ (e ^3^)	0.61, 0.54

Computer programs: *APEX2* and *SAINT* (Bruker, 2014 ▸), *SHELXT* (Sheldrick, 2015*a* ▸), *SHELXL2014* (Sheldrick, 2015*b* ▸), *OLEX2* (Dolomanov *et al.*, 2009 ▸), *Mercury* (Macrae *et al.*, 2008 ▸) and *publCIF* (Westrip, 2010 ▸).

## supplementary crystallographic information

### Crystal data

**Table:**

(C_4_H_7_N_2_)_2_[CoCl_4_]	*F*(000) = 1480
*M**_r_* = 366.96	*D*_x_ = 1.633 Mg m^−^^3^
Monoclinic, *C*2/*c*	Ga *K*α radiation, λ = 1.34139 Å
*a* = 26.847 (3) Å	Cell parameters from 9758 reflections
*b* = 7.9029 (8) Å	θ = 5.1–61.0°
*c* = 15.0938 (14) Å	µ = 10.45 mm^−^^1^
β = 111.184 (6)°	*T* = 100 K
*V* = 2986.0 (5) Å^3^	Block, clear light blue
*Z* = 8	0.23 × 0.12 × 0.06 mm

### Data collection

**Table:**

Bruker Venture Metaljet diffractometer	3435 independent reflections
Radiation source: Metal Jet, Gallium Liquid Metal Jet Source	3037 reflections with *I* > 2σ(*I*)
Helios MX Mirror Optics monochromator	*R*_int_ = 0.063
Detector resolution: 10.24 pixels mm^-1^	θ_max_ = 60.9°, θ_min_ = 3.1°
ω and φ scans	*h* = −34→34
Absorption correction: multi-scan (*SADABS*; Krause *et al.*, 2015)	*k* = −9→10
*T*_min_ = 0.392, *T*_max_ = 0.752	*l* = −19→19
27212 measured reflections	

### Refinement

**Table:**

Refinement on *F*^2^	Primary atom site location: structure-invariant direct methods
Least-squares matrix: full	Hydrogen site location: mixed
*R*[*F*^2^ > 2σ(*F*^2^)] = 0.042	H atoms treated by a mixture of independent and constrained refinement
*wR*(*F*^2^) = 0.102	*w* = 1/[σ^2^(*F*_o_^2^) + (0.0316*P*)^2^ + 16.8591*P*] where *P* = (*F*_o_^2^ + 2*F*_c_^2^)/3
*S* = 1.10	(Δ/σ)_max_ = 0.001
3435 reflections	Δρ_max_ = 0.61 e Å^−^^3^
188 parameters	Δρ_min_ = −0.54 e Å^−^^3^
0 restraints	

### Special details

**Table:**

Experimental. X-ray crystallographic data for I were collected from a single-crystal sample, which was mounted on a loop fiber. Data were collected using a Bruker Venture diffractometer equipped with a Photon 100 CMOS Detector, a Helios MX optics and a Kappa goniometer. The crystal-to-detector distance was 4.0 cm, and the data collection was carried out in 1024 *x* 1024 pixel mode.
Geometry. All e.s.d.'s (except the e.s.d. in the dihedral angle between two l.s. planes) are estimated using the full covariance matrix. The cell e.s.d.'s are taken into account individually in the estimation of e.s.d.'s in distances, angles and torsion angles; correlations between e.s.d.'s in cell parameters are only used when they are defined by crystal symmetry. An approximate (isotropic) treatment of cell e.s.d.'s is used for estimating e.s.d.'s involving l.s. planes.

### Fractional atomic coordinates and isotropic or equivalent isotropic displacement parameters (Å^2^)

**Table:**

	*x*	*y*	*z*	*U*_iso_*/*U*_eq_	
N3	0.55652 (11)	0.8138 (4)	0.22412 (19)	0.0239 (6)	
N4	0.55734 (11)	0.8201 (4)	0.08322 (19)	0.0233 (6)	
C5	0.55675 (14)	0.5281 (4)	0.1500 (3)	0.0280 (7)	
H5A	0.5891	0.4842	0.1993	0.042*	
H5B	0.5555	0.4895	0.0876	0.042*	
H5C	0.5252	0.4865	0.1614	0.042*	
C6	0.55747 (12)	0.7154 (4)	0.1528 (2)	0.0221 (6)	
C7	0.55565 (14)	0.9825 (5)	0.1998 (2)	0.0252 (7)	
C8	0.55618 (14)	0.9864 (5)	0.1105 (2)	0.0263 (7)	
N1	0.68727 (11)	0.1769 (4)	0.2752 (2)	0.0250 (6)	
N2	0.68983 (11)	−0.0922 (4)	0.2666 (2)	0.0247 (6)	
C1	0.68961 (15)	0.0217 (5)	0.4219 (3)	0.0331 (8)	
H1A	0.6906	0.1353	0.4485	0.050*	
H1B	0.7215	−0.0415	0.4606	0.050*	
H1C	0.6576	−0.0376	0.4219	0.050*	
C2	0.68836 (12)	0.0349 (4)	0.3234 (2)	0.0246 (7)	
C3	0.68876 (14)	0.1398 (5)	0.1869 (3)	0.0282 (7)	
C4	0.69070 (14)	−0.0300 (5)	0.1817 (3)	0.0275 (7)	
Co1	0.62832 (2)	0.50011 (6)	0.45110 (3)	0.01829 (13)	
Cl1	0.57795 (3)	0.73678 (10)	0.44067 (5)	0.02206 (17)	
Cl2	0.56998 (3)	0.28410 (10)	0.38769 (5)	0.02368 (17)	
Cl3	0.67944 (3)	0.43980 (10)	0.60249 (5)	0.02279 (17)	
Cl4	0.68208 (3)	0.54327 (10)	0.36494 (5)	0.02248 (17)	
H8	0.5553 (17)	1.078 (6)	0.068 (3)	0.039 (12)*	
H4A	0.6916 (16)	−0.099 (6)	0.132 (3)	0.036 (11)*	
H7	0.5553 (16)	1.071 (5)	0.241 (3)	0.031 (10)*	
H3A	0.6871 (17)	0.228 (6)	0.145 (3)	0.043 (12)*	
H3	0.5586 (16)	0.778 (5)	0.275 (3)	0.029 (11)*	
H4	0.5609 (16)	0.789 (5)	0.037 (3)	0.033 (11)*	
H2	0.6908 (17)	−0.186 (6)	0.281 (3)	0.037 (13)*	
H1	0.6852 (19)	0.264 (6)	0.295 (3)	0.043 (14)*	

### Atomic displacement parameters (Å^2^)

**Table:**

	*U*^11^	*U*^22^	*U*^33^	*U*^12^	*U*^13^	*U*^23^
N3	0.0226 (13)	0.0358 (17)	0.0158 (12)	−0.0017 (12)	0.0099 (10)	0.0007 (12)
N4	0.0237 (13)	0.0321 (16)	0.0172 (13)	−0.0005 (11)	0.0110 (11)	−0.0005 (11)
C5	0.0249 (16)	0.0278 (19)	0.0326 (18)	−0.0001 (14)	0.0119 (14)	0.0006 (14)
C6	0.0162 (14)	0.0306 (18)	0.0204 (14)	−0.0001 (12)	0.0075 (11)	0.0014 (13)
C7	0.0256 (16)	0.0278 (18)	0.0238 (16)	−0.0006 (13)	0.0107 (13)	−0.0034 (14)
C8	0.0245 (16)	0.0314 (19)	0.0240 (16)	0.0008 (13)	0.0100 (13)	0.0030 (14)
N1	0.0223 (14)	0.0212 (16)	0.0307 (15)	0.0002 (11)	0.0086 (11)	−0.0024 (12)
N2	0.0255 (14)	0.0171 (15)	0.0333 (15)	−0.0001 (11)	0.0129 (12)	0.0035 (12)
C1	0.0303 (18)	0.041 (2)	0.0306 (19)	0.0018 (16)	0.0141 (15)	0.0032 (16)
C2	0.0164 (14)	0.0280 (18)	0.0293 (17)	−0.0002 (12)	0.0082 (12)	0.0008 (14)
C3	0.0257 (17)	0.0275 (19)	0.0316 (18)	−0.0033 (14)	0.0106 (14)	0.0015 (15)
C4	0.0289 (17)	0.0282 (19)	0.0275 (17)	−0.0011 (14)	0.0128 (14)	−0.0025 (14)
Co1	0.0212 (2)	0.0198 (2)	0.0161 (2)	0.00038 (17)	0.00940 (17)	0.00017 (16)
Cl1	0.0257 (4)	0.0242 (4)	0.0185 (3)	0.0057 (3)	0.0107 (3)	0.0011 (3)
Cl2	0.0286 (4)	0.0248 (4)	0.0202 (3)	−0.0047 (3)	0.0118 (3)	−0.0035 (3)
Cl3	0.0228 (4)	0.0257 (4)	0.0190 (3)	0.0007 (3)	0.0066 (3)	0.0031 (3)
Cl4	0.0280 (4)	0.0224 (4)	0.0228 (3)	0.0000 (3)	0.0160 (3)	0.0008 (3)

### Geometric parameters (Å, º)

**Table:**

N3—C6	1.336 (4)	N1—H1	0.76 (5)
N3—C7	1.381 (5)	N2—C2	1.330 (5)
N3—H3	0.79 (4)	N2—C4	1.381 (4)
N4—C6	1.336 (4)	N2—H2	0.77 (5)
N4—C8	1.381 (5)	C1—H1A	0.9800
N4—H4	0.78 (4)	C1—H1B	0.9800
C5—H5A	0.9800	C1—H1C	0.9800
C5—H5B	0.9800	C1—C2	1.479 (5)
C5—H5C	0.9800	C3—C4	1.346 (5)
C5—C6	1.481 (5)	C3—H3A	0.93 (5)
C7—C8	1.354 (5)	C4—H4A	0.94 (4)
C7—H7	0.94 (4)	Co1—Cl1	2.2799 (9)
C8—H8	0.96 (5)	Co1—Cl2	2.2803 (9)
N1—C2	1.332 (5)	Co1—Cl3	2.2506 (8)
N1—C3	1.380 (5)	Co1—Cl4	2.2907 (8)
			
C6—N3—C7	110.6 (3)	C2—N2—C4	110.1 (3)
C6—N3—H3	124 (3)	C2—N2—H2	123 (3)
C7—N3—H3	126 (3)	C4—N2—H2	127 (3)
C6—N4—C8	110.4 (3)	H1A—C1—H1B	109.5
C6—N4—H4	123 (3)	H1A—C1—H1C	109.5
C8—N4—H4	126 (3)	H1B—C1—H1C	109.5
H5A—C5—H5B	109.5	C2—C1—H1A	109.5
H5A—C5—H5C	109.5	C2—C1—H1B	109.5
H5B—C5—H5C	109.5	C2—C1—H1C	109.5
C6—C5—H5A	109.5	N1—C2—C1	126.6 (3)
C6—C5—H5B	109.5	N2—C2—N1	106.6 (3)
C6—C5—H5C	109.5	N2—C2—C1	126.8 (3)
N3—C6—N4	106.1 (3)	N1—C3—H3A	119 (3)
N3—C6—C5	126.9 (3)	C4—C3—N1	106.4 (3)
N4—C6—C5	126.9 (3)	C4—C3—H3A	134 (3)
N3—C7—H7	123 (3)	N2—C4—H4A	124 (3)
C8—C7—N3	106.3 (3)	C3—C4—N2	106.7 (3)
C8—C7—H7	130 (3)	C3—C4—H4A	130 (3)
N4—C8—H8	121 (3)	Cl1—Co1—Cl2	106.55 (3)
C7—C8—N4	106.6 (3)	Cl1—Co1—Cl4	108.56 (3)
C7—C8—H8	132 (3)	Cl2—Co1—Cl4	110.57 (3)
C2—N1—C3	110.2 (3)	Cl3—Co1—Cl1	111.89 (3)
C2—N1—H1	123 (4)	Cl3—Co1—Cl2	110.00 (3)
C3—N1—H1	127 (4)	Cl3—Co1—Cl4	109.25 (3)
			
N3—C7—C8—N4	0.0 (4)	N1—C3—C4—N2	0.6 (4)
C6—N3—C7—C8	−0.1 (4)	C2—N1—C3—C4	0.1 (4)
C6—N4—C8—C7	0.1 (4)	C2—N2—C4—C3	−1.1 (4)
C7—N3—C6—N4	0.1 (4)	C3—N1—C2—N2	−0.8 (4)
C7—N3—C6—C5	−177.8 (3)	C3—N1—C2—C1	177.3 (3)
C8—N4—C6—N3	−0.1 (4)	C4—N2—C2—N1	1.1 (4)
C8—N4—C6—C5	177.8 (3)	C4—N2—C2—C1	−176.9 (3)

### Hydrogen-bond geometry (Å, º)

**Table:**

*D*—H···*A*	*D*—H	H···*A*	*D*···*A*	*D*—H···*A*
N1—H1···Cl4	0.76 (5)	2.46 (5)	3.220 (3)	177 (5)
N2—H2···Cl4^i^	0.77 (5)	2.54 (5)	3.282 (3)	163 (4)
N3—H3···Cl1	0.79 (4)	2.39 (4)	3.166 (3)	165 (4)
N4—H4···Cl2^ii^	0.78 (4)	2.42 (5)	3.198 (3)	175 (4)
C4—H4*A*···Cl3^iii^	0.94 (4)	2.73 (4)	3.428 (4)	132 (3)
C3—H3*A*···Cl3^ii^	0.93 (5)	2.70 (5)	3.535 (4)	151 (4)
C8—H8···Cl1^iv^	0.96 (5)	2.65 (5)	3.575 (4)	160 (3)
C7—H7···Cl2^v^	0.94 (4)	2.69 (4)	3.617 (4)	168 (3)
C5—H5*A*···Cl4	0.98	2.86	3.738 (4)	149
C5—H5*C*···Cl2^vi^	0.98	2.88	3.771 (4)	152
C1—H1*B*···Cl4^vii^	0.98	2.95	3.804 (4)	146
C1—H1*C*···Cl1^i^	0.98	2.87	3.840 (4)	169

Symmetry codes: (i) *x*, *y*−1, *z*; (ii) *x*, −*y*+1, *z*−1/2; (iii) *x*, −*y*, *z*−1/2; (iv) *x*, −*y*+2, *z*−1/2; (v) *x*, *y*+1, *z*; (vi) −*x*+1, *y*, −*z*+1/2; (vii) −*x*+3/2, −*y*+1/2, −*z*+1.

## References

[bb1] Adams, C. J., Kurawa, M. A., Lusi, M. & Orpen, A. G. (2008). *CrystEngComm*, **10**, 1790–1795.

[bb2] Bruker (2014). *APEX2* and *SAINT*. Bruker AXS Inc., Madison, Wisconsin, USA.

[bb3] Dolomanov, O. V., Bourhis, L. J., Gildea, R. J., Howard, J. A. K. & Puschmann, H. (2009). *J. Appl. Cryst.* **42**, 339–341.

[bb4] Groom, C. R. & Allen, F. H. (2014). *Angew. Chem. Int. Ed.* **53**, 662–671. 10.1002/anie.201306438 24382699

[bb5] Issaoui, F., Baklouti, Y., Dharhi, E., Zouari, F. & Valente, M. A. (2015). *J. Supercond. Nov. Magn.* **28**, 10.1007/s10948-015-3057-y.

[bb6] Krause, L., Herbst-Irmer, R., Sheldrick, G. M. & Stalke, D. (2015). *J. Appl. Cryst.* **48**, 3–10. 10.1107/S1600576714022985 PMC4453166 26089746

[bb7] Macrae, C. F., Bruno, I. J., Chisholm, J. A., Edgington, P. R., McCabe, P., Pidcock, E., Rodriguez-Monge, L., Taylor, R., van de Streek, J. & Wood, P. A. (2008). *J. Appl. Cryst.* **41**, 466–470.

[bb8] Mghandef, M. & Boughzala, H. (2015). *Acta Cryst.* E**71**, 555–557. 10.1107/S2056989015007707 PMC4420110 25995878

[bb9] Sheldrick, G. M. (2015*a*). *Acta Cryst.* A**71**, 3–8.

[bb10] Sheldrick, G. M. (2015*b*). *Acta Cryst.* C**71**, 3–8.

[bb11] Westrip, S. P. (2010). *J. Appl. Cryst.* **43**, 920–925.

[bb12] Zhang, H., Fang, L. & Yuan, R. (2005). *Acta Cryst.* E**61**, m677–m678.

